# A Novel Surgical Technique for Total Knee Arthroplasty Using the Femoral Trochlear Bisector as a New Landmark: Technical Description and Early Clinical Results

**DOI:** 10.3390/jcm15020404

**Published:** 2026-01-06

**Authors:** Maurilio Marcacci, Alberto Favaro, Gregorio Alberto, Luca Alberti, Simonetta Resta, Tommaso Bonanzinga, Francesco Iacono

**Affiliations:** 1 Department of Biomedical Sciences, Humanitas University, Via Rita Levi Montalcini 4, Pieve Emanuele, 20072 Milan, Italy; maurilio.marcacci@gmail.com (M.M.); gregorio.alberto@humanitas.it (G.A.); lucabiz98@gmail.com (L.A.); tommaso.bonanzinga@hunimed.eu (T.B.); 2 IRCCS Humanitas Research Hospital, Via Manzoni 56, Rozzano, 20089 Milan, Italy; alberto.favaro@humanitas.it (A.F.); francesco.iacono.bo@gmail.com (F.I.)

**Keywords:** knee, alignment, osteoarthritis, radiography, arthroplasty, replacement, trochlear groove bisector

## Abstract

**Background/Objectives**: This study introduces and evaluates a novel surgical technique for total knee arthroplasty (TKA) that uses the trochlear groove bisector (TGB) as an anatomical landmark to achieve kinematic alignment of the femoral component in the coronal plane. The aim of the present retrospective observational analysis was to assess the reproducibility and accuracy of this approach and to report preliminary clinical outcomes. **Methods**: Twenty-eight TKA procedures were performed using the TGB-guided alignment technique. Preoperative planning and postoperative assessments were conducted on long-leg weight-bearing radiographs to measure the agreement between planned and achieved alignment, analysed using Bland–Altman statistics. Functional outcomes were evaluated at 12 months with the Knee Society Score (KSS), Forgotten Joint Score (FJS), and patient satisfaction. **Results**: The mean difference between planned and post-operative mLDFA was −0.3° ± 0.65°, with a root mean square error (RMSE) of 0.7°, demonstrating high accuracy and reproducibility. Postoperative outcomes showed mean KSS (knee = 89.6, function = 91.4), FJS = 69.6 ± 12, mean flexion = 124.6°, and mean HKA = 178°. Ninety percent of patients reported being satisfied or very satisfied at 12 months. **Conclusions**: The TGB-based technique offers a reliable, easily applicable method for restoring native femoral alignment in TKA using standard instrumentation. It allows accurate prediction of postoperative alignment and achieves favourable early functional outcomes. While currently limited to moderate varus deformities, future developments integrating 3D or CT-based planning may extend its applicability to more complex cases.

## 1. Introduction

In recent years, the total number of knee arthroplasties has increased dramatically, spiking by 30% since 2012 and accounting for nearly 680,000 cases in 2022 in Europe alone. Most of these procedures are associated with osteoarthritis (OA) mainly driven by ageing and obesity [1,2]. In this context, anatomical alignment has represented one of the earliest alignment strategies described for total knee arthroplasty (TKA). As originally reported by Hungerford and Krackow in 1985, this approach was designed to reproduce native femoral alignment by applying a fixed 3° femoral valgus and 3° tibial varus, thereby orienting the femoral component parallel to the posterior condylar axis. However, due to the technical difficulties in performing the varus cut on the tibia in precise and reproducible way, this method was abandoned [3].

With the aim of achieving knee stability, the neutral mechanical alignment philosophy was introduced [4,5]. This was based on the principle that the mechanical axis (MA), which passes through the centre of the knee, facilitates a balanced mediolateral load distribution, thereby reducing implant wear and the risk of component loosening. Despite providing good long-term implant survivorship [6], 10% of cases yield unsatisfactory patient functional outcomes in terms of pain, effusion, and instability [7,8,9]. A new technique known as kinematic alignment (KA) was later introduced by Howell et al. in 2014 [10] as an alternative approach through the restoration of the native pre-arthritic limb joint alignments and knee laxity [11,12,13,14]. The approach foresees a “true knee resurfacing”, involving symmetric anatomical cuts on the femur and tibia condyles to compensate for bone and cartilage loss. The resections are tailored to match the thickness of the prosthetic components and are subsequently verified using calliper measurements. This so-called “callipered unrestricted” (or “measured unrestricted”) KA technique relies exclusively on bone cuts accuracy to achieve the pre-arthritic alignment, and seeks to maintain the knee’s ligamentous stability and kinematics. The “unrestricted” KA technique itself and the use of a calliper make this approach technically demanding. Moreover, this methodology can result in extreme alignment outliers compared to mechanical alignment TKA, potentially leading to reduced implant survivorship [15]. To address this concern, the “restricted” kinematic alignment (rKA) was developed, which imposes limits on the coronal alignment of femoral and tibial cuts [16].

Building on these approaches, functional alignment has emerged as a strategy that addresses the limitation of mechanical alignment and KA by restoring joint line orientation and limb alignment within a safe range that minimizes soft tissue imbalance, thus reducing the need for extensive ligament releases and optimizing knee kinematics [17]. Despite the great patient satisfaction and functional outcomes demonstrated by this technique, particularly when robotic-assisted systems are employed [18], additional clinical investigations are required to establish long-term implant survivorship [19].

In an attempt to restore the knee’s native alignment without laborious procedural steps, our group introduced in a previous radiographic study the trochlear groove bisector (TGB) as a new anatomical landmark, identified on anteroposterior weight-bearing radiographs of healthy lower limbs [20]. The bisector exhibits a perpendicular orientation to the healthy femoral joint line and, consequently, perpendicular to the knee primary femoral axis. Therefore, when performing TKA in OA patients where the native joint line is no longer identifiable due to the effect of OA, surgeons can fully restore the femoral joint line of the native knee by resecting the distal femur perpendicularly to the bisector of the trochlear groove, and, in doing so, aligning the femoral component to the primary femoral axis in the coronal plane.

In contrast to the use of other alignment techniques, the use of TGB as anatomical landmark for positioning the femoral component in the coronal plane allows preoperative prediction of the final alignment preserving the soft tissue envelope. The intervention can also be executed using standard surgical instrumentation, without requiring callipers, nor patient-specific guides or advanced systems such as navigators or robots.

In this study, we aim to evaluate and highlight the early clinical outcomes of a pilot cohort of patients who underwent TKA guided by TGB, introducing and assessing this novel alignment philosophy in a clinical setting. We hypothesize that the use of TGB-guided bone resections can restore the native pre-arthritic alignment, inherently achieving correct ligament tensioning and knee stability, consequently improving surgery outcomes.

## 2. Materials and Methods

This study was conducted on pre-operative and post-operative radiographic data from patients with symptomatic osteoarthritis classified as Kellgren–Lawrence grade (KLG) 3, who had undergone TKAs with the TGB-guided alignment approach. All interventions had been performed at the Humanitas Research Hospital between February 2021 and November 2022 and resulted in the application of a posterior stabilized (PS) TKA (Attune, Depuy Italia, Rome, Italy). Use of patient data was in agreement with prior written consent by the patients authorizing handling of their anonymized personal data for scientific purposes. Since the study involved anonymized radiographic imaging alone with no clinical data, no ethical committee approval was needed.

Of the initial sample of radiographic imaging analysed using TraumaCad Neo software (Brainlab AG, Munich, Germany), 28 X-rays associated with 28 knees belonging to 28 patients were deemed suitable for the study, having met the following inclusion criteria: (i) X-ray of patients with a primary diagnosis of KLG = 3 symptomatic primary knee OA or post-traumatic arthritis; (ii) X-ray of patients with a pre-operative Knee Society Score (KSS) Knee score of >25 and <75. X-rays were excluded if they featured limbs featuring valgus deformity, with severe deformities (including varus deformity > 15°, trochlear dysplasia, or altered Q-angles), or had confounding radiographic elements and images with incorrect limb rotation, based on patellar position and lesser trochanter profile.

The demographic data and baseline clinical data of the patients whose X-rays were included in the study are reported in Table 1.

### 2.1. Radiographic Evaluation and Preoperative Planning

Preoperative planning was performed using weight-bearing long-leg coronal X-rays. The radiographic assessment included anteroposterior (AP) and lateral views of the knee to be operated and full-limb AP X-rays with the knee extended and the patella facing forward, which ensured the correct positioning of the limb.

**Figure 1:**
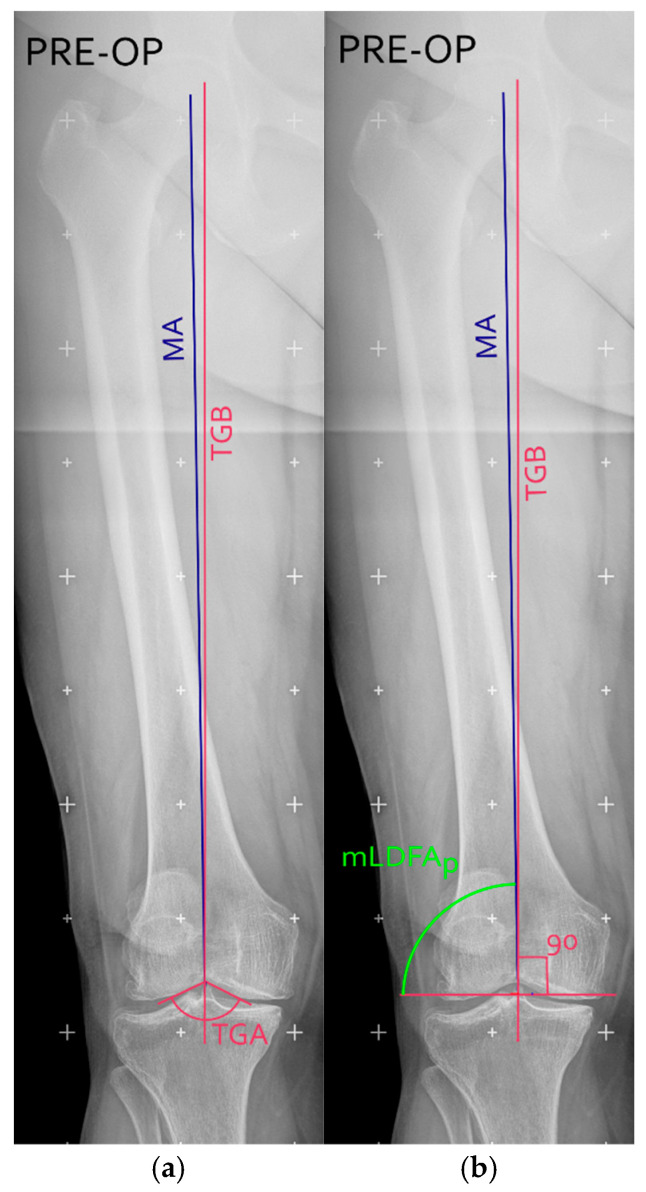
Zoomed versions of the right knee in a pre-operative long-leg coronal X-ray, with the representation of the mechanical axis (MA, blue line); the trochlear groove bisector (TGB, purple line); trochlear groove angle (TGA) (**a**) and planned mechanical distal femoral (mLDFA_p_, green angle) (**b**). Figure created with Inkscape (version 1.4.3, The Inkscape Project, Boston, MA, USA).

After the identification of bone landmarks, hip-knee angle (HKA), anatomical axis (AA), mechanical axis (MA), anatomical mechanical angle (AMA), and mechanical lateral distal femoral angles (mLDFA) were measured using standardized techniques to guide bone resections and implant positioning [20]. Also, the TGB was measured on long-leg coronal X-rays, as previously described [20]. In brief, the trochlear groove angle (TGA) was determined by drawing two tangential lines along the medial and lateral aspects of the trochlear groove from the radiographic apical midpoint of the intercondylar groove (see Figure 1a). The TGB was then drawn as the bisector of the TGA (purple vertical line in Figure 1a).

The distal femoral cut (purple horizontal line in Figure 1b) was planned perpendicular to the TGB. Once the distal femoral cut was identified, the MA was drawn and the planned mLDFA (mLDFA_p_) was defined as the angle between the planned cut and the MA [21].

Lastly, the angle between MA and TGB, which was expressed as Delta (∆), was computed (see Figure 1b). When TGB was medial to MA, ∆ took positive values; conversely, when TGB was lateral to MA, ∆ took negative values.

The tibial cut was planned parallel to the joint line and was equal in thickness to the tibial component, with correction for cartilage wear. The planned mechanical medial proximal tibial angle (mMPTA) was set parallel to the femoral cut, ensuring a maximum varus limit of 3°.

### 2.2. Surgical Technique

The TKAs were performed following a medial parapatellar approach with a tourniquet. Soft tissue dissection was limited to a conservative exposure of the medial tibial plateau, without release of the superficial medial collateral ligament. Bone resections were carried out using mechanical instruments. Following the removal of tibial and femoral osteophytes, the anterior cruciate ligament was sacrificed.

A “femur-first” technique was used for bone resections. The distal femoral cut was executed so to replicate the mLDFA_p_.

**Figure 2:**
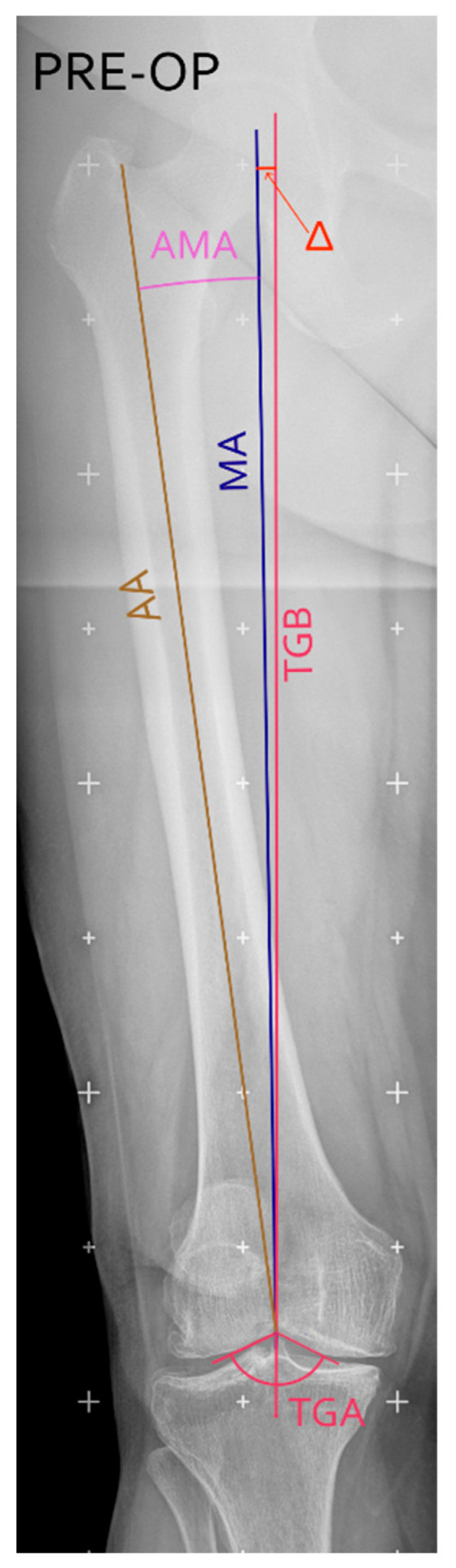
In case the intramedullary guide was used to perform the femoral cut, the angle on pre-operative long-leg coronal X-rays was calculated summing the anatomical mechanical angle (AMA, in pink), defined as the angle between the MA (blue line) and the anatomical axis (AA, ochre yellow line), and ∆ (in red), defined as the angle between MA and the TGB (purple line). Figure created with Inkscape (version 1.4.3, The Inkscape Project, Boston, MA, USA).

In the event an intramedullary guide was used, the resection had to be opportunely adjusted considering the preoperatively calculated AMA angle (see Figure 2), according to the following rule:

intramedullary guide angle = AMA + ∆

Medial and lateral gaps were assessed using spacer blocks with the knee in full extension, ensuring coronal limb alignment within the 0–3° range.

Axial alignment was set according to the posterior condylar line to maintain alignment with the cylindrical/posterior condylar axis. This alignment could be adjusted within 1–3° of external rotation to prevent internal rotation of the trochlear groove, with strict reference to Whiteside’s line [22]. The femoral component size was determined as small as possible [23], using the posterior condyles as reference and selecting the size that prevents femoral overhang, anterior notching, mediolateral overhang, and patellofemoral joint overstuffing.

The proximal tibial cut was performed using an extramedullary guide, replicating the varus angle preoperatively planned in the coronal plane and providing a posterior slope of 0° in the sagittal plane. Axial alignment of the tibial component was carried out according to Akagi’s line [24].

**Figure 3:**
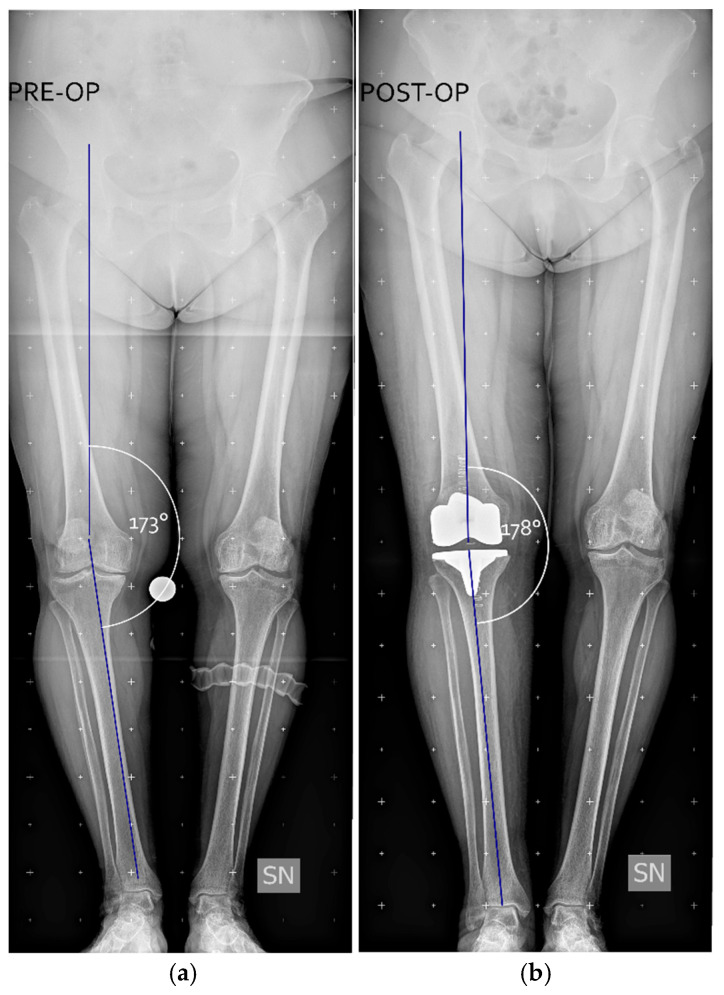
Example of a clinical case from the data set. Panel (**a**) shows a preoperative weight-bearing full leg X-rays with a hip-knee angle (HKA, in white), defined as the angle between the femur and tibia mechanical axes (blue lines), measuring 173°. Panel (**b**) shows the postoperative correction, with the HKA brought to 178°. Figure created with Inkscape (version 1.4.3, The Inkscape Project, Boston, MA, USA).

After positioning the prosthesis trials, medial and lateral gaps were reassessed using spacer blocks in both extension and flexion, aiming for symmetrical residual laxity of 1–2 mm in both compartments, with a HKA angle of 176–180°. Figure 3 shows a clinical case where a pre-operative varus aliment of 7° (HKA = 173°) was brought post-operatively to a varus of 2° (HKA = 178°).

In those cases where the knee was balanced but excessively tight in both extension and flexion, a tibial recut was performed. The approach favoured preservation of the native femoral anatomy and the knee’s natural flexion axis—essentially making this approach a “femur-driven” technique. If tightness concerned flexion alone, the posterior tibial slope was increased up to 3°; otherwise, if tightness concerned extension alone, an additional distal femoral cut was made.

In cases of femorotibial soft-tissue imbalance in both flexion and extension, the proximal tibia was recut to restore balance while maintaining overall limb alignment within 4° of varus (i.e., HKA in the range 180–176°).

When corrections were needed only in flexion, the femoral component was rotated within 1–3° in the axial plane to adjust the flexion gap. A slight lateral laxity (1–1.5 mm greater than the medial compartment) was considered acceptable. In cases of imbalance in extension, the distal femoral cut was adjusted by introducing up to 3° of valgus in the coronal plane to balance the extension gap.

To preserve joint line integrity and minimize mid-flexion instability, over-resection of the distal femur was avoided [25]. Conversely, under-resection was prevented to reduce dependence on thinner polyethylene inserts, which could compromise flexion stability [26]. Valgus cuts of the proximal tibia were also avoided, as they increased the risk of revision in constitutional varus patients [27]. When bone recuts proved insufficient to restore balance, selective soft tissue releases were considered.

### 2.3. Postoperative Evaluation

**Figure 4:**
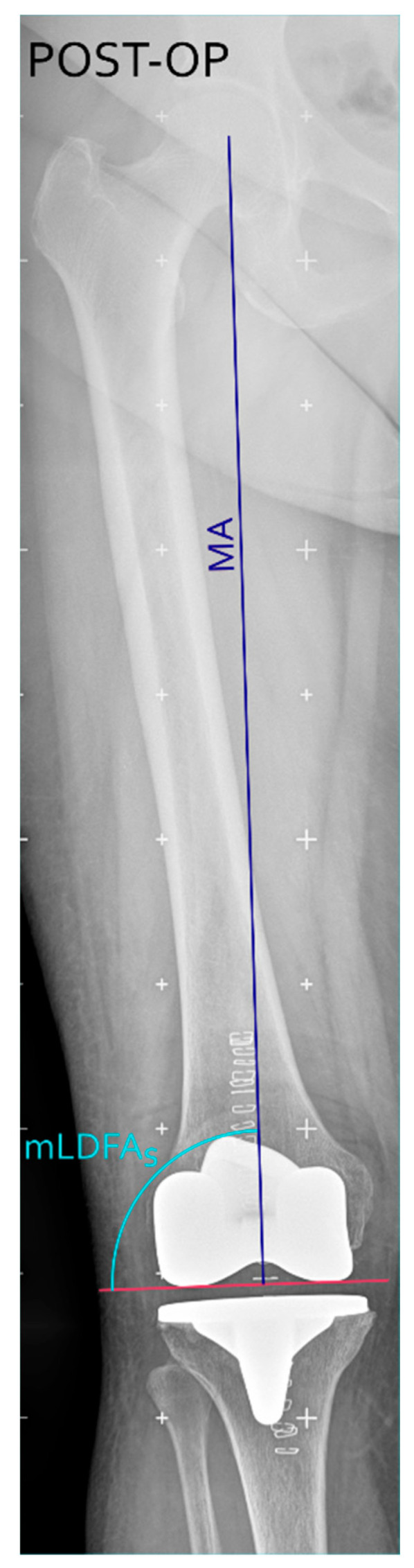
Postoperative long-leg coronal X-ray, showing the postoperative component positioning with the surgical mechanical lateral distal femoral angle (mLDFA_s_, in light blue) defined as the angle between the distal femoral cut (purple horizontal line) and the MA (blue line). Figure created with Inkscape (version 1.4.3, The Inkscape Project, Boston, MA, USA).

To quantitatively assess the accuracy and reproducibility of the proposed technique for distal femoral resection, we considered original mechanical lateral distal femoral angles (mLDFA_o_), mLDFA_p_, surgical mechanical lateral distal femoral angle (mLDFA_s_, i.e., the post-operative mLDFA measured on long leg X-rays) (see Figure 4) and the resection error (defined as the difference between mLDFA_p_ and mLDFA_s_) as follows.

Resection error = mLDFA_p_ − mLDFA_s_

All patients had undergone postoperative clinical and radiological follow-ups at 1, 3 and 12 months after surgery. The 12-month postoperative assessment included physical parameters, such as flexion, flexion contracture, and HKA angle, and patient-reported outcomes, such as the Forgotten Joint Score (FJS) [28], the Knee Society Score (KSS knee, KSS function) and patient satisfaction level, using a 5-point Likert scale. This scale was selected as it provides a simple, reliable, and widely validated method for capturing subjective postoperative perceptions, facilitating comparison with existing literature; it also ensures consistency with standard patient-reported outcome approaches. Descriptive statistics including mean, standard deviation, minimum, and maximum values are reported for each parameter. The Root Mean Square Error (RMSE) was also calculated for resection error to assess global surgical accuracy. The agreement between the planned and surgical distal femoral angles (mLDFA_p_ vs. mLDFA_s_) was evaluated through a Bland–Altman analysis. Limits of Agreement defining the range within which 95% of the differences between the two meaurements (mLDFA_p_ and mLDFA_s_) was calculated as:

Limits of Agreements: Mean value ± Standard Deviation × 1.96

This statistical method allows quantification of systematic bias and the clinical acceptability of the differences, which cannot be determined through correlation analysis alone.

## 3. Results

Mean values of the mLDFA_o_, planned mLDFA_p_ and mLDFA_s_ from the 28 X-rays are reported in Table 2.

mLDFA_o_, measured on pre-operative radiographs, mLDFA_p_, defined during preoperative planning, and mLDFA_s_, measured on postoperative radiographs, showed comparable values. Moreover, the mLDFA_s_ exhibited a reduced standard deviation compared with both preoperative and planned values. To quantitatively assess the accuracy and reproducibility of the proposed technique, statistical analysis of the resection error was performed and is reported below in Table 3.

**Figure 5:**
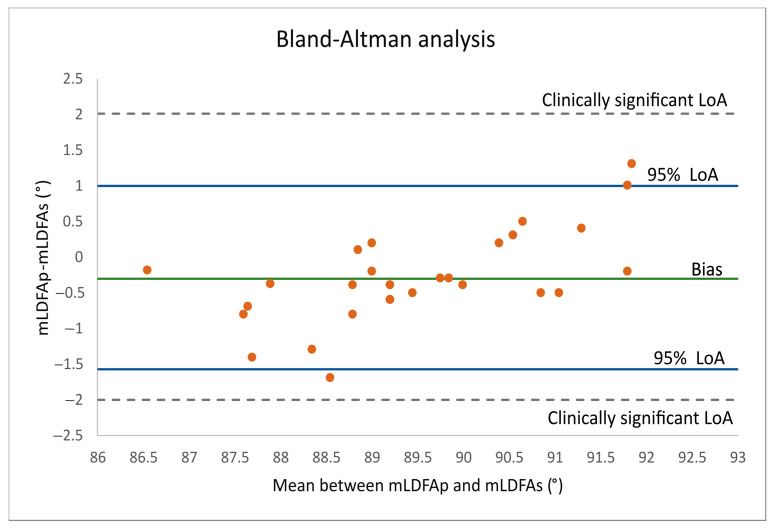
Bland–Altman plot assessing the agreement between mLDFA_p_ and mLDFA_s_. The *x*-axis represents the mean of the two measurements, while the *y*-axis displays their difference. Each bullet point represents a single knee case. The mean difference is depicted by the green line at −0.3°, whereas the upper and blue lines represent the 95% Limits of Agreement.

The difference between the planned and the postoperative surgical angles (mLDFA_p_ − mLDFA_s_) demonstrates a RMSE of 0.70°, indicating a low overall deviation from the planned target. The mean difference was −0.3° ± 0.65, with values ranging from −1.7° to 1.3°. The slightly negative mean suggests a minimal tendency toward undercorrection relative to the planned alignment, although the magnitude of this deviation is clinically negligible. The agreement between mLDFA_p_ and mLDFA_s_ was further assessed through Bland–Altman analysis, as reported in Figure 5.

The Bland–Altman analysis demonstrated good agreement between mLDFA_p_ and mLDFA_s_. The mean difference (bias) was small and negative (approximately −0.3°), indicating that mLDFA_p_ slightly underestimated mLDFA_s_. The 95% LoA values were narrow and entirely within the predefined clinically acceptable limits (±2°).

### Clinical Outcomes

Clinical outcomes were evaluated postoperatively using standard scores (KSS, the FJS) and physical parameters (flexion, flexion contracture, and HKA angle) and are reported in Table 4.

The Knee Society Scores show high mean values for both the knee component (KSS Knee: 89.6 ± 8.9) and the functional component (KSS Function: 91.4 ± 12.4), indicating overall good to excellent clinical and functional results. Also, the observed FJS values (mean value of 69.9 ± 12) are indicative of favourable to excellent clinical outcomes, with limited inter-patient variability. Range of motion (ROM) analysis reveals a mean postoperative knee flexion of 124.6° ± 10 (range 100–140), while flexion contracture is minimal (0.2° ± 1.2; range 0–5), suggesting effective restoration of joint functionality. Radiographic evaluation of lower limb alignment shows a mean HKA angle of 178° ± 2 (range 176–180), with no cases of extreme alignment observed.

Moreover, patient satisfaction was collected at 12 months’ follow-up using a 5-point Likert scale. Table 5 below shows that 90% of patients were satisfied with outcome of surgery at 12 months’ follow up.

## 4. Discussion

The present pilot study proposes TGB as a valid tool for aligning the femoral component in the coronal plane in TKA, without significantly modifying the mLDFA. This is in agreement with the acknowledgement that, differently from the cartilage (which is subjected to wearing), the bone surface is marginally influenced by the presence of the OA, and thus the mLDFA is not expected to change significantly between mLDFA_o_ and mLDFA_s_. Indeed, mLDFA_o_, mLDFA_p_ and mLDFA_s_ calculated on 28 patients was consistent. A small RMSE (0.71°) between the preoperative and postoperative mLDFA evaluated on weight-bearing long-leg X-rays supports the TGB-based approach for predicting the post-operative alignment with good accuracy and reproducibility. Such a small RMSE aligns with errors commonly observed in TKAs procedures and measured in literature linked to the use of the intramedullary guide (0.66–0.79°) [29]. The agreement between planned and achieved alignment values was provided by the robustness of the Bland–Altman analysis.

Mean values from the Bland–Altman analysis between the planned and postoperative mLDFA measurements were close to 0°:−0.3° (green line in the Bland–Altman plot of Figure 5), indicates a slight although almost negligible trend toward varization. Also, most data points fall within the LoA, showing a consistent and uniform distribution around zero especially in the interval between 88.5° and 91°, indicating an excellent level of agreement between the planned and executed distal femoral resections. In the range between 86° to 88.5°, the majority of data points are positioned below the bias line, while from 91° to 93°, several data points lie above zero, thus indicating a trend in reducing the obliquity (both in varus and valgus directions) when the planned MLDFA lies significantly far from neutral. Although two cases fell outside the LoA, they still remained within the clinically significant LoA of ±2° established for the difference between mLDFA_p_ and mLDFA_s_.

Differently from Howell’s kinematic alignment approach, a key advantage of this surgical approach is that it does not require an estimation of the cartilage wear. By relying on bony landmarks that are less prone to be altered by the pathological bone condition, this method avoids inaccuracies related to osteoarthritic cartilage loss, which may otherwise affect surgical planning.

Additionally, this method has demonstrated high versatility since it can be performed using both standard intramedullary guides commonly used in surgical practice without the need for advanced technologies such as robotic or navigation systems, although it remains compatible with these technologies, when available. This flexibility allows for broad clinical adoption across different surgical settings.

The approach also enables one to perform preoperative planning on weight-bearing radiographs, the imaging method routinely used in standard clinical practice. This guarantees that the technique can be easily implemented with conventional instrumentation, without requiring additional resources or specialized equipment.

Clinical results at 12 months of follow-up reported an improved for ROM, function and pain between pre- and post-operative values. In total, 90% of patients were satisfied. The high FJS [30] suggests a restoration of the native alignment and the achieving of a joint ligament balancing throughout the arc of knee motion, without modifying ligamentous structures. This positive results indicate that patients experience a natural-feeling knee, with minimal awareness of the prosthesis during daily activities, reflecting a high degree of joint–prosthesis integration. We hypothesize that the favourable patient outcomes can be also attributed to a biomechanical aspect brought by using the TGB as a reference for the femoral component alignment. Future studies will need to investigate in depth whether the TGB aligns with the quadriceps force vector of the leg extension mechanism, thus providing a physiological movement of the trochlea over the femoral component that may explain the favourable outcomes obtained by patient treated with this technique.

### Study Limitations

Although this surgical technique offers significant advantages, one shortcoming of the current procedure is its use in limited to moderate varus knees (<15°). Indeed, greater femoral deformities could have hindered an optimal and reliable identification of the trochlear bisector on radiographs.

Another limitation is represented by the quality of X-ray acquisition, which needed to be high quality in order to allow the proper recognition of the anatomical landmarks on the radiograph. Such limitations could, however, be addressed in the future by using computer navigation and robotic systems, which use Computed Tomography (CT) scan for planning. CT-based 3D reconstructions, by providing three-dimensional images allow for the analysis of the trochlear groove at different depths, offering a more precise assessment compared to the two-dimensional evaluation performed with X-rays. This could allow to expand the applicability of the method to cases where the trochlear groove is difficult to identify on radiographs due to limitations of X-ray imaging (such as poor image quality or incorrect femoral component rotation) or in the presence of complex femoral anatomies and/or deformities.

Another limitation of this study is the small sample size of patients and the short follow-up period for clinical evaluation. However, the aim of this work was to evaluate the reproducibility and effectiveness of a TKA surgical technique that employs a new landmark (trochlear groove bisector) to perform the femoral resection, and not of presenting a case series or a study cohort.

Finally, the use of traditional intramedullary guides to perform the distal femoral resection might also be considered as a limitation in terms of accuracy replicating the preoperative plan (although limited RMSE has been evidenced between the planned and performed resections), but this could be easily overcome by using new technologies like sensor-based guides or navigation systems. These systems could offer real-time intraoperative feedback on resection angles, alignment, and implant positioning, thereby helping to reduce surgical errors and enhance the accuracy and reproducibility of femoral and tibial cuts, potentially lowering the RMSE.

## 5. Conclusions

In the present study, we describe a new surgical technique based on the trochlear groove bisector (TGB) as a new radiological landmark in TKA. Our findings support the validity and reliability of the TGB as a reference for guiding surgeons in restoring native kinematic alignment and achieving balanced ligamentous tension throughout the range of knee motion. By providing a reproducible anatomical landmark, the TGB-based approach may also help mitigate mid-flexion instability, which is frequently associated with patient dissatisfaction and suboptimal functional outcomes. Despite this technique being currently limited to moderate varus deformities, future developments involving CT-based reconstruction and new technologies like sensor-based guides or navigation systems may allow its application in cases of more pronounced deviations in the coronal plane. Moreover, the TGB-based approach is compatible with both conventional instrumentation and more advanced technological solutions, which may further enhance the precision and reproducibility of femoral component positioning.

Future studies will include randomized control trials to evaluate the clinical outcomes, long-term implant survival, and potential functional benefits of this technique across a wider spectrum of TKA candidates and alignment strategies.

Overall, the TGB represents a promising tool for improving surgical accuracy, optimizing knee kinematics, and potentially enhancing patient satisfaction following TKA.

## Figures and Tables

**Table 1:** Patient demographics.

Parameter	Value
Mean age at surgery, years (range)	73 (63–79)
Sex distribution	11 females (39%), 17 males (61%)
Mean varus deformity	10°
Hip-knee-ankle (HKA) angle (range)	166–175°
Body mass index (BMI) (range)	22.2–38.75 kg/m^2^

**Table 2:** Statistics of original mechanical lateral distal femoral angle (mLDFA_o_), planned mechanical lateral distal femoral angle (mLDFA_p_), surgical mechanical lateral distal femoral angle (mLDFA_s_).

	MEAN	(±) SD	MIN	MAX
**mLDFA_o_**	89.4	1.65	86	92.3
**mLDFA_p_**	89.3	1.63	86.5	92.5
**mLDFA_s_**	89.6	1.22	86.7	91.9

**Table 3:** Statistical evaluation of the resection error.

	RMSE	MEAN	(±) SD	MIN	MAX
**mLDFA_p_ − mLDFA_s_**	0.70	−0.3	0.65	−1.7	1.3

**Table 4:** Values of postoperative conditions of the treated patients in terms of Knee Society Score (KSS), knee and function, Forgotten Joint Score (FJS) and flexion, flexion contracture and hip–knee angle degrees.

	MEAN	(±) SD	MIN	MAX
**KSS Knee**	89.6	8.9	53	100
**KSS Function**	91.4	12.4	35	100
**FJS**	69.9	12	20	100
**Flexion (°)**	124.6	10	100	140
**Flexion Contracture (°)**	0.20	1.2	0	5
**HKA Angle (°)**	178	2	176	180

**Table 5:** Patient satisfaction levels assessed 12 months postoperatively.

Level of Satisfaction	Very Satisfied	Satisfied	Neutral	Unsatisfied	Very Unsatisfied
**Patients *n* (%)**	16 (57.1%)	9 (32.1%)	3 (10.8%)	0 (0%)	0 (0%)

## Data Availability

The data that support the findings of this study are available from the corresponding author upon reasonable request.

## Abbreviations

The following abbreviations are used in this manuscript:

AA	Anatomical Axis
AMA	Anatomical Mechanical Angle
AP	Anteroposterior
CT	Computed Tomography
FJS	Forgotten Joint Score
HKA	hip-knee-ankle angle
KA	Kinematic Alignment
KLG	Kellgren–Lawrence grade
KSS	Knee Society Score
MA	Mechanical Axis
mLDFA	Mechanical Lateral Distal Femoral Angle
mLDFA_o_	Original Mechanical Lateral Distal Femoral Angle
mLDFA_p_	Planned Mechanical Lateral Distal Femoral Angle
mLDFA_s_	Surgical Mechanical Lateral Distal Femoral Angle
mMPTA	Mechanical Medial Proximal Tibial Angle
OA	Osteoarthritis
PS	Posterior Stabilized
rKA	Restricted Kinematic Alignment
RMSE	Root Mean Square Error
ROM	Range of Motion
TGA	Trochlear Groove Angle
TGB	Trochlear Groove Bisector
TKA	Total Knee Arthroplasty
